# Standardization of MR Electrical Properties Tomography: A Guideline From the ISMRM Electro‐Magnetic Tissue Properties Study Group

**DOI:** 10.1002/jmri.70230

**Published:** 2026-01-16

**Authors:** Stefano Mandija, Alessandro Arduino, Chuanjiang Cui, Patrick Fuchs, Ilias I. Giannakopoulos, Yusuf Ziya Ider, Kyu‐Jin Jung, Nitish Katoch, Ulrich Katscher, Dong‐Hyun Kim, Riccardo Lattanzi, Thierry Meerbothe, Freddy Odille, Karin Shmueli, Paul Soullié, Khin Khin Tha, Luca Zilberti, Cornelis A. T. van den Berg

**Affiliations:** ^1^ Computational Imaging Group for MR Therapy and Diagnostic, Department of Radiotherapy University Medical Center Utrecht Utrecht the Netherlands; ^2^ Istituto Nazionale di Ricerca Metrologica Torino Italy; ^3^ Department of Electrical and Electronic Engineering Yonsei University Seoul Republic of Korea; ^4^ Department of Medical Physics and Biomedical Engineering University College London London UK; ^5^ Bernard and Irene Schwartz Center for Biomedical Imaging, Department of Radiology New York University Grossman School of Medicine New York New York USA; ^6^ Department of Biomedical Engineering Baskent University Ankara Turkey; ^7^ Department of Electrical Engineering and Computer Sciences University of California Berkeley California USA; ^8^ Philips Healthcare Seoul South Korea; ^9^ Philips Innovative Technologies Hamburg Germany; ^10^ Center for Advanced Imaging Innovation and Research (CAI^2^R), Department of Radiology New York University Grossman School of Medicine New York New York USA; ^11^ IADI U1254, INSERM and Université de Lorraine Nancy France; ^12^ Global Center for Biomedical Science and Engineering, Hokkaido University Faculty of Medicine Kita‐ku, Sapporo Japan

**Keywords:** conductivity, MR‐EPT standardization, permittivity

## 1 Introduction

MR Electrical Properties Tomography (MR‐EPT) is an MRI‐based technique that allows non‐invasive measurement of the electrical properties of human tissue (i.e., electrical conductivity *σ* and relative permittivity *ε*_*r*_).

Over the past two decades, numerous methods have been published on how to reconstruct such properties from MRI data, as reported in a comprehensive review [1].

However, published in vivo results show a large variability in reconstructed conductivity and relative permittivity values, which may arise from the different data processing approaches, as also observed in the first MR‐EPT reconstruction challenge [2, 3].

Looking through the previous MR‐EPT literature, we realize that it is often difficult to replicate the presented results or compare them across publications. There are several factors that contribute to this lack of standardization:

- Lack of methodological details reported in published work;
- Large variability in presenting the results, both qualitatively in figures and quantitatively through metrics;
- Unavailability of common data and evaluation metrics

This guideline complements two other MR‐EPT guidelines, that is, on phantoms [4] and acquisitions [5], and aims to standardize the reporting of methods and results in scientific publications to support reproducibility and comparability of new acquisition and reconstruction methods for MR‐EPT, while indirectly improving MR‐EPT reconstruction quality, study validity, and efficacy.

## 2 Reporting of Methods

### 2.1 Phantoms

Reporting phantom recipes, geometry, and reference values of electrical properties is of paramount importance. For details on what to report in publications, we refer the reader to the guideline on phantoms for MR‐EPT [4].

### 2.2 Simulations

Most MR‐EPT methods are developed and tested using simulated data, for example, as the ones reported in the ADEPT database [6]. In addition to the simulation software, the following items should be reported or previous publications reporting them should be cited:

- RF coils: setup, dimensions, transmission/reception channels, polarization type, schematic diagram of the simulated coil;
- Simulation: simulation type, grid resolution (mm), frequency (MHz) of the excitation ports and phase offset, accuracy (convergence of the solver);
- Noise: noise level, definition of Signal‐to‐Noise Ratio (SNR) adopted, and how noise was added to the data;
- Synthetic contrast‐weighted images: generation method (e.g., parameter settings such as repetition time, echo time, T1, T2 when using Bloch equations).

### 2.3 Measurements

Imaging techniques and parameters have been suggested in the MR‐EPT acquisition guideline [5]. Overall, the following information should be provided:

- MRI system: MRI scanner type, field strength, vendor, coil setup;
- Type of sequence (e.g., Spin Echo, Gradient Echo), sampling strategy (e.g., Cartesian, Radial, Spiral), and whether the used sequences are for phase‐based EPT (i.e., measuring the transceive phase), magnitude‐based EPT (i.e., measuring the magnitude of B_1_^+^), or complex EPT (aiming to measure both the magnitude and phase of the B_1_^+^ field);
- Acquisition parameters: resolution (mm), field‐of‐view (mm), image orientation, 2D/multi‐slice/3D, repetition time (ms), echo time (ms), flip angle (°), radiofrequency (RF) pulse characteristics, bandwidth (Hz) per voxel, number of repetitions (averages), type(s) of acceleration technique(s) and corresponding acceleration factors, oversampling factors, type of shimming, acquisition time (s);
- SNR and how this was computed, for example, using the single image NEMA approach [7], where the standard deviation of the noise may be derived from within the area of signal if imaging filters automatically remove background noise in air. We recommend measuring SNR so that simulations can be made at a similar SNR to allow proper comparison;
- Report coil combination methods and how the transceive phase was computed, including phase correction methods, unwrapping, and post‐processing.

### 2.4 Reconstructions

Most MR‐EPT methods rely on three steps: pre‐processing, reconstruction of EPs, and post‐processing:

- Pre/post‐processing—the following should be reported:
  ○ Type of filters applied before and/or after EPs reconstruction, including filters kernel size (mm), weights, constraints to tissue‐specific regions.
- Reconstruction method—the following should be reported:
  ○ Type of reconstruction, for example, direct, inverse, differential/integral‐based, image‐based, deep‐learning, 2D, 3D;
  ○ Type of output, that is, conductivity (S/m) only or both conductivity (S/m) and relative permittivity (‐);
  ○ The reconstruction algorithm or reference(s) to a publication(s) explaining it;
  ○ Imaging data used for the reconstruction, for example, transceive phase for phase‐only MR‐EPT;
  ○ Assumptions on B1‐fields, field‐components, or EPs, such as the local homogeneity assumption, and on the use of the signal magnitude, for example, for constraining the reconstruction to specific regions with the same signal intensity;
  ○ Input parameters for example, kernel or patch size (mm) and weights;
  ○ Reconstruction time (min:s), including training time (min:s) for deep‐learning methods and computing hardware specifications;
  ○ If applicable, size of training and validation datasets.
- Tissue structure—If a priori knowledge on tissue structure was used either for the pre/post‐processing steps or EPs reconstruction, the following information should be provided:
  ○ Tissue structure and/or tissue boundary information used;
  ○ How this was derived, for example, segmentation, image contrast, boundary detection methods;
  ○ Software packages and parameters used to obtain this information.

## 3 Reporting of Results

To define a standardized way of reporting both MR‐EPT images and quantitative values, a Delphi process, similar to that used to standardize color‐maps for MR relaxometry [8], was performed within the Electro‐Magnetic Tissue Properties (EMTP) Study Group of the International Society of Magnetic Resonance in Medicine (ISMRM), including both clinical and technical expertise as well as industry representatives. The process relied on a panel of up to 25 experts from the EMTP Study Group who were asked to answer four questionnaires between January and April 2025. Details on the process and questionnaires are provided in Data S1.

The following statements about color‐maps, ranges, data availability, and metrics reached consensus. Alongside these consensus statements, recommendations are provided to facilitate benchmarking of new reconstruction methods.

### 3.1 Color Maps

1. A color‐map should contain a specific color, clearly distinguishable from all the other colors in the color‐map, to indicate regions of the image having unknown electrical properties values.
2. The proposed color‐maps should be available on common processing platforms, such as Python and Matlab.
3. The proposed color‐maps should be made available for free.
4. Each quantitative image must be displayed in conjunction with a color‐bar with adequately readable numbers.
5. The proposed color‐maps should be as perceptually linear as possible.
6. The proposed color‐maps should be as perceptually linear as possible also when viewed by people with deuteranopia (red/green blindness).
7. The proposed color‐maps should be as perceptually linear as possible also when viewed by people with color blindness
8. The proposed color‐maps should be as perceptually linear as possible also when converted to greyscale (printed copy)
9. Conductivity and relative permittivity should have two different color‐maps.
10. Conductivity and relative permittivity maps should have a black background (air).

**Recommendation:** Since a consensus on the color‐maps to be used was not reached, we recommend using color‐maps reported in Crameri et al. [9], that are in agreement with the statements that reached consensus. To facilitate comparison between scientific publications, we recommend using Lipari for conductivity and Navia for relative permittivity. If necessary, these maps may be adapted to include a color clearly distinguishable from the others, dedicated to regions of unknown EPs. A consensus about how to display areas with erroneous EPs values (either negative or too high) was not reached. However, based on the trend of answers, negative EPs may be displayed with the color of the background. Questions were not formulated on how to display EPs with unrealistically high values. However, we recommend displaying these with the color of the upper bound of the color‐map.

### 3.2 Ranges

1. For scientific publications, the min‐max range applied to a color‐map should not be fixed to certain values but should always be freely adaptable.
2. In publications, the min‐max ranges can be freely adaptable (authors' freedom); but maps using predefined (standardized) min‐max ranges should be displayed (e.g., in supporting materials of publications) to facilitate comparison between papers. These ranges will therefore be defined and will be different based on frequency and anatomical area.
3. If zoomed‐in images are made to highlight some details, the color‐bar of the full image should be maintained. Hence, the min‐max values remain the same as the full image and are not adjusted to the min‐max values of the zoomed‐in image.
4. The range for brain conductivity should be fixed: 0–2.5 S/m at 3 T (128 MHz).

**Recommendation:** The suggested ranges for relative permittivity did not reach consensus, while the range of conductivity that reached consensus applies to the brain at 3 T (128 MHz). To facilitate comparison between scientific publications, we recommend using 0–2.5 S/m for conductivity and 30–100 for relative permittivity, both at 1.5 T (64 MHz) and 3 T (128 MHz).

### 3.3 Data Availability and Metrics

1. An example dataset should be provided and made available online.
2. One simulated dataset (with and without noise) (3 T–128 MHz) on a healthy brain model should be available.
3. One simulated dataset (with and without noise) (3 T–128 MHz) on a brain model with a tumor inclusion should be available.
4. The available dataset should contain the ground truth electrical property values as reference.
5. One dataset measured at 3 T (128 MHz) on a phantom should be available.
6. Such phantoms should be: (1) a sphere (diameter = 12 cm) and (2) a cylinder (diameter = 12 cm, length = 12 cm); reference electrical properties values measured using probes during phantom construction should be provided.
7. Alongside the example datasets, analysis scripts to be run on the reconstructed EPs maps should be provided.
8. The analysis scripts can be written in multiple languages, but should include MATLAB and Python.
9. The following should be computed in these scripts for each tissue of interest: mean, standard deviation, median, interquartile ranges, absolute error, and relative error.
10. The following should be computed in these scripts for the whole volume (all tissues where electrical properties were reconstructed): NRMSE.
11. The masks used for these analyses should contain different levels of erosion and the results should be computed and reported for these different levels as in the EPT challenge (0–2–4 voxels erosion at boundaries) [2, 3].

**Recommendation:** Based on the above consensus statements, to facilitate benchmarking of new methods on common data using a standardized reporting structure and analysis scripts, we recommend:

1. Testing new reconstruction methods on the following datasets provided here:
  - Simulation at 3 T (128 MHz)—healthy brain model—without and with noise
  - Simulation at 3 T (128 MHz)—brain model with tumor inclusion—without and with noise
  - Measurement at 3 T (128 MHz)—cylindrical phantom
  - Measurement at 3 T (128 MHz)—spherical phantom
2. Running the analysis scripts provided here on the reconstructed electrical property maps on the above datasets.
3. Reporting the results generated by the provided analysis scripts using the available templates.
  Datasets, analysis scripts to be run on reconstructed electrical properties maps using the provided input datasets, and reporting templates (including an example of a standardized report) are available here: https://doi.org/10.5281/zenodo.17879937

If other datasets and metrics are used, the following information should be provided:

- Types of metrics and specifications on how they were computed;
- How data were masked and/or Regions‐of‐Interest (ROI) were computed, including the number of voxels, volume in mm^3^ of the masked region or ROI;
- If (tissue) boundary erosion was performed: type of erosion function, 2D vs. 3D erosion, number of voxels eroded at boundary;
- How unrealistic conductivity and relative permittivity values were handled (e.g., negative values, positive outliers, erroneous values at tissue boundaries were discarded or set to zero);
- For in vivo dataset: report basic subject characteristics (age, sex).

## 4 Open Science

We encourage researchers to publish datasets and code alongside their publications. A link to these repositories can be added to the community website: EMTPhub.org.

## Supporting information


**Data S1:** Delphi process information and rounds.
**Table S1:** Results Round 1.
**Table S2:** Results Round 2.
**Table S3:** Results Round 3.
**Table S4:** Results Round 4.

## Data Availability

Datasets, analysis scripts to be run on reconstructed electrical properties maps using the provided input datasets, and reporting templates are available here: https://doi.org/10.5281/zenodo.17879937.
