# Supplementary material for: Standardization of MR Electrical Properties Tomography: A Guideline From the ISMRM Electro‐Magnetic Tissue Properties Study Group
Source: J Magn Reson Imaging. 2026 Jan 16;63(4):1204–7. doi: 10.1002/jmri.70230 (PMC12963806; doi:10.1002/jmri.70230)
Supplement: Supplementary file 1 — Data S1: Delphi process information and rounds. Table S1: Results Round 1. Table S2: Results Round 2. Table S3: Results Round 3. Table S4: Results Round 4. [file JMRI-63-1204-s001.docx]

**Supporting Material 1**

**DELPHI PROCESS**

The process relied on a panel of up to 25 experts from the ISMRM EMTP Study Group, who were asked to answer four questionnaires between January and April 2025.

After each survey round, a summary of the results was compiled and provided to the panel of experts, allowing them to revise their answers in light of the replies from other panel members.

Similarly to Fuderer et al. (1), a 9-point Likert scale was used, ranging from ‘fully disagree’ to ‘fully agree’.

Based on the comments of earlier rounds, some questions were either rephrased or removed.

For each round, the questions and scores are reported in Supporting Tables 1-4. For these reports, the 9-point Likert scale was summarized to a 3-point Likert scale: “disagree” for scores 1, 2 and 3; “neutral” for scores 4, 5 and 6; “agree” for scores 7, 8 and 9, where, according to Diamond et al. (2), a 75% consensus threshold was applied. This means that it was considered a consensus whenever the sum of scores 7, 8, or 9 (or 1, 2, or 3) reached 75% of all responses in that round. This threshold of 75% agreement, set a priori, was used as it is a common choice in Delphi processes (1, 2).

For each round, the statements that reached consensus are highlighted in bold.

**Supplementary table 1:** results Round 1.

| **Round 1 – 21 responders** | **Agreement** | **Consensus** | **Note** |
| --- | --- | --- | --- |
| **Color-maps** |  |  |  |
| Conductivity and relative permittivity should get two different color-maps. | 67% |  |  |
| Conductivity and relative permittivity maps should have a black background (air). | 71% |  |  |
| **A color-map should contain a specific color, clearly distinguishable from all the other colors, to indicate regions of the image having unknown electrical properties values.** | 76% | Yes |  |
| A color-map should contain a specific color, clearly distinguishable from all the other colors, to indicate regions of the image having negative electrical properties values. | 67% |  | rephrased |
| **The proposed color-maps should be available on common processing platforms like Python, Matlab.** | 90% | Yes |  |
| **The proposed color-maps should be made available for free.** | 81% | Yes |  |
| **Each quantitative image must be displayed in conjunction with a color-bar with adequately readable numbers.** | 86% | Yes |  |
| **The proposed color-maps should be as perceptually linear as possible.** | 86% | Yes |  |
| The proposed color-maps should be as perceptually linear as possible, also when viewed by people with deuteranopia (red/green blindness). | 62% |  |  |
| The proposed color-maps should be as perceptually linear as possible, also when viewed by people with color blindness. | 57% |  |  |
| The proposed color-maps should be as perceptually linear as possible, also when converted to greyscale (printed copy). | 67% |  |  |
| We should use a (warm) color-map like Lipari for conductivity (doi/10.1002/mrm.30290). | 62% |  | rephrased |
| We should use a (cold) color-map like Navia for relative permittivity (doi/10.1002/mrm.30290). | 52% |  | rephrased |
| We should use exactly Lipari and Navia respectively for conductivity (left) and relative permittivity (right) as for T1 and T2 mapping (doi/10.1002/mrm.30290). | 43% |  | rephrased |
| We should use different color-maps than the ones above for conductivity and relative permittivity, e.g., rainbow, although they might not adhere with scientific standards as reported in doi:10.1038/s41467-020-19160-7 | 29% |  | rephrased |
| **Ranges** |  |  |  |
| For scientific works, the min-max range applied to a color-map should not be fixed to certain values, but should always be freely adaptable. Note that this means that two different papers showing conductivity maps, e.g. in the brain, may adopt two different max values, hence the color related to conductivity in the white matter may be different between the two papers, which may limit direct comparison between color-maps. | 52% |  | slightly rephrased |
| For clinical works, the min-max range applied to a color-map should not be fixed to certain values, but should always be freely adaptable. Note that this means that two different papers showing conductivity maps, e.g. in the brain, may adopt two different max values, hence the color related to conductivity in the white matter may be different between the two papers, which may limit direct comparison between color-maps. | 29% |  | slightly rephrased |
| The range for conductivity should be fixed: 0-3 S/m to facilitate comparison between publications. | 48% |  |  |
| The range for relative permittivity should be fixed: 1-100 to facilitate comparison between publications. | 52% |  |  |
| If zoomed-in images are made to highlight some details, the color bar of the full image should be maintained. Hence, the min-max values remain the same ones of the full image and are not adjusted to the ones of the zoomed-in image. | 57% |  |  |
| **Data and Metrics** |  |  |  |
| **Example datasets, available online, for people to test their methods should be provided.** | 90% | Yes |  |
| **A link to these datasets should be provided as supplementary material of the standardization guideline.** | 95% | Yes |  |
| The example datasets should be a subset of the ones already used for the EPT challenge. | 38% |  |  |
| One simulated dataset (with and without noise) (3 T - 128 MHz) on a cylinder should be available. | 62% |  |  |
| **One simulated dataset (with and without noise) (**3 T - **128 MHz) on a brain model should be available.** | 86% | Yes |  |
| One simulated dataset at 3 T (128 MHz) on a brain model + tumor inclusion (with and without noise) should be available. | 67% |  |  |
| **The available dataset should contain the ground truth electrical property values as reference.** | 100% | Yes |  |
| **One measured dataset at** 3 T (**128 MHz) on a phantom should be available.** | 76% | Yes |  |
| Such phantom should be a sphere (diameter=12cm); reference electrical properties from constructions should be provided. | 48% |  |  |
| The same type of measurements should also be provided at 1.5 T (64 MHz). | 43% |  |  |
| Alongside the example datasets, analyses scripts to be run on the reconstructed EP maps should be provided. | 67% |  |  |
| The analysis scripts should be written in Matlab. | 57% |  |  |
| The analysis scripts should be written in Python. | 48% |  |  |
| The analysis scripts should be supplied as precompiled executables for most major operating systems. | 57% |  |  |
| When possible, people should provide the results from the above scripts in the supplementary materials of their publications to allow direct comparisons between methodologies. | 67% |  |  |
| A standardized template to be used as supplementary materials in publications for such type of reporting, e.g., one image of the reconstructed EP maps, and a table with the quantitative results from the analysis scripts should be provided. | 57% |  |  |
| The standardized template and scripts should be provided as supplementary material of the standardization guideline. | 67% |  |  |
| **A link to the standardization guideline and materials should be reported in the EMTP hub website.** | 86% | Yes |  |
| **The following should be computed in these scripts for each tissue of interest: [Mean]** | 95% | Yes |  |
| **The following should be computed in these scripts for each tissue of interest: [Standard Deviation]** | 95% | Yes |  |
| The following should be computed in these scripts for each tissue of interest: [Median] | 62% |  |  |
| The following should be computed in these scripts for each tissue of interest: [Interquartile range] | 52% |  |  |
| The following should be computed in these scripts for each tissue of interest: [Absolute error] | 71% |  |  |
| The following should be computed in these scripts for each tissue of interest: [Relative error] | 62% |  |  |
| The following should be computed in these scripts for each tissue of interest: [Histograms] | 57% |  |  |
| The following should be computed in these scripts for the whole volume: [NRMSE] | 67% |  |  |
| The following should be computed in these scripts for the whole volume: [SSIM] | 52% |  |  |
| The masks used for these analyses should not be eroded despite the risk that errors at tissue boundaries will skew the quantitative values. | 33% |  |  |
| The masks used for these analyses should contain different levels of erosion, and the results should be computed and reported for these different levels like in the EPT challenge (0-2-4 voxels erosion at boundaries). | 62% |  |  |

**Supplementary table 2:** results Round 2.

| **Round 2 – 19 responders** | **Agreement** | **Consensus** | **Note** |
| --- | --- | --- | --- |
| **Color-maps** |  |  |  |
| **Conductivity and relative permittivity should get two different color-maps.** | 84% | Yes |  |
| **Conductivity and relative permittivity maps should have a black background (air).** | 79% | Yes |  |
| Regions of negative electrical properties values should have the same color of the: [background] | 32% |  |  |
| Regions of negative electrical properties values should have the same color of the: [lower limit of the colorbar being this >=0] | 68% |  |  |
| **The proposed color-maps should be as perceptually linear as possible, also when viewed by people with deuteranopia (red/green blindness).** | 89% | Yes |  |
| **The proposed color-maps should be as perceptually linear as possible, also when viewed by people with color blindness.** | 89% | Yes |  |
| **The proposed color-maps should be as perceptually linear as possible, also when converted to greyscale (printed copy).** | 89% | Yes |  |
| In publications, the color-maps may have different colors than the recommended ones (authors' freedom); but maps using the recommended colors should also be displayed (e.g., in supporting materials of publications) to facilitate comparison between papers. | 63% |  |  |
| The color-map for conductivity should be: [exactly Lipari] | 37% |  |  |
| The color-map for conductivity should be: [warm-like Lipari] | 42% |  |  |
| The color-map for conductivity should be: [different from Lipari, although it might not adhere to scientific standards as reported in doi:10.1038/s41467-020-19160-7.] | 21% |  |  |
| The color-map for relative permittivity should be: [exactly Navia] | 26% |  |  |
| The color-map for relative permittivity should be: [cold-like Navia] | 42% |  |  |
| The color-map for relative permittivity should be: [different from Navia, although it might not adhere to scientific standards as reported in doi:10.1038/s41467-020-19160-7.] | 16% |  |  |
| **Ranges** |  |  |  |
| For scientific works, the min-max range applied to a color-map should not be fixed to certain values, but should always be freely adaptable. Note that this means that two different papers showing conductivity maps, e.g. in the brain, may adopt two different max values, hence the color related to conductivity in the white matter may be different between the two papers. This may limit direct comparison between color-maps but can also help highlight details. | 74% |  |  |
| For clinical works, the min-max range applied to a color-map should not be fixed to certain values, but should always be freely adaptable. Note that this means that two different papers showing conductivity maps, e.g. in the brain, may adopt two different max values, hence the color related to conductivity in the white matter may be different between the two papers. This may limit direct comparison between color-maps but can also help highlight details. | 42% |  |  |
| In publications, the min-max ranges can be freely adaptable (authors' freedom); but maps using predefined min-max ranges should be displayed (e.g., in supporting materials of publications) to facilitate comparison between papers. These ranges will be therefore defined and will be different based on frequency and anatomical area. | 68% |  |  |
| If zoomed-in images are made to highlight some details, the color bar of the full image should be maintained. Hence, the min-max values remain the same ones of the full image and are not adjusted to the ones of the zoomed-in image. | 74% |  |  |
| The range for brain conductivity should be fixed: 0-2.5 S/m at 3 T (128 MHz) to facilitate comparison between publications. | 68% |  |  |
| The range for brain relative permittivity should be fixed: 40-100 at 3 T (128 MHz) to facilitate comparison between publications. | 42% |  |  |
| **Data and Metrics** |  |  |  |
| The example datasets should be a subset of the ones already used for the EPT challenge. | 58% |  |  |
| One simulated dataset (with and without noise) (3 T - 128 MHz) on a cylinder should be available. | 68% |  |  |
| One simulated dataset at 3 T (128 MHz) on a brain model + tumor inclusion (with and without noise) should be available. | 68% |  |  |
| One measured dataset at 3 T (128 MHz) on a phantom should be available [reached consensus]. Statement: Such phantom should be a sphere (diameter=12cm) and reference electrical properties values from phantom construction should be provided. | 47% |  |  |
| The same type of measurements should also be provided at 1.5 T (64MHz). | 37% |  | removed |
| Alongside the example datasets, analyses scripts to be run on the reconstructed EP maps should be provided. | 68% |  |  |
| The analysis scripts can be written in multiple languages, but should include Matlab. | 63% |  |  |
| The analysis scripts can be written in multiple languages, but should include Python. | 53% |  |  |
| The analysis scripts should be supplied as precompiled executables for most major operating systems. The source code will also be provided. | 53% |  |  |
| When possible, people should provide the results from the above scripts in the supplementary materials of their publications to allow direct comparisons between methodologies. | 63% |  |  |
| A standardized template to be used as supplementary materials in publications for such type of reporting, e.g., one image of the reconstructed EP maps, and a table with the quantitative results from the analysis scripts should be provided. | 63% |  |  |
| The standardized template and scripts should be provided as supplementary material of the standardization guideline. | 58% |  |  |
| The following should be computed in these scripts for each tissue of interest: [Median] | 63% |  |  |
| The following should be computed in these scripts for each tissue of interest: [Interquartile range] | 68% |  |  |
| The following should be computed in these scripts for each tissue of interest: [Absolute error] | 68% |  |  |
| The following should be computed in these scripts for each tissue of interest: [Relative error] | 68% |  |  |
| The following should be computed in these scripts for each tissue of interest: [Histograms] | 42% |  | removed |
| The following should be computed in these scripts for the whole volume: [NRMSE] | 74% |  |  |
| The following should be computed in these scripts for the whole volume: [SSIM] | 37% |  | removed |
| The masks used for these analyses should not be eroded despite the risk that errors at tissue boundaries will skew the quantitative values. | 32% |  |  |
| The masks used for these analyses should contain different levels of erosion, and the results should be computed and reported for these different levels like in the EPT challenge (0-2-4 voxels erosion at boundaries). | 74% |  |  |

**Supplementary table 3:** results Round 3.

| **Round 3 – 19 responders** | **Agreement** | **Consensus** | **Note** |
| --- | --- | --- | --- |
| **Color-maps** |  |  |  |
| Regions of negative electrical properties values should have the same color as the: [background] | 53% |  |  |
| Regions of negative electrical properties values should have the same color as the: [lower limit of the colorbar, this being >=0] | 58% |  |  |
| In publications, the color-maps may have different colors than the recommended ones (authors' freedom); but maps using the recommended colors should also be displayed (e.g., in supporting materials of publications) to facilitate comparison between papers. | 58% |  |  |
| The color-map for conductivity should be: [exactly Lipari] | 58% |  |  |
| The color-map for conductivity should be: [warm-like Lipari] | 16% |  |  |
| The color-map for conductivity should be: [different from Lipari, although it might not adhere to scientific standards as reported in doi:10.1038/s41467-020-19160-7.] | 32% |  | removed |
| The color-map for relative permittivity should be: [exactly Navia] | 58% |  |  |
| The color-map for relative permittivity should be: [cold-like Navia] | 16% |  |  |
| The color-map for relative permittivity should be: [different from Navia, although it might not adhere to scientific standards as reported in doi:10.1038/s41467-020-19160-7.] | 26% |  | removed |
| **Ranges** |  |  |  |
| For scientific works, the min-max range applied to a color-map should not be fixed to certain values, but should always be freely adaptable. Note that this means that two different papers showing conductivity maps, e.g. in the brain, may adopt two different max values, hence the color related to conductivity in the white matter may be different between the two papers. This may limit direct comparison between color-maps but can also help highlight details. | 68% |  |  |
| For clinical works, the min-max range applied to a color-map should not be fixed to certain values, but should always be freely adaptable. Note that this means that two different papers showing conductivity maps, e.g. in the brain, may adopt two different max values, hence the color related to conductivity in the white matter may be different between the two papers. This may limit direct comparison between color-maps but can also help highlight details. | 68% |  |  |
| In publications, the min-max ranges can be freely adaptable (authors' freedom); but maps using predefined min-max ranges should be displayed (e.g., in supporting materials of publications) to facilitate comparison between papers. These ranges will be therefore defined and will be different based on frequency and anatomical area. | 68% |  |  |
| **If zoomed-in images are made to highlight some details, the color bar of the full image should be maintained. Hence, the min-max values remain the same as the full image and are not adjusted to the min-max values of the zoomed-in image.** | 79% | Yes |  |
| The range for brain conductivity should be fixed: 0-2.5 S/m at 3 T (128 MHz) to facilitate comparison between publications. | 68% |  |  |
| The range for brain relative permittivity should be fixed: 30-100 at 3 T (128 MHz) to facilitate comparison between publications. | 47% |  |  |
| **Data and Metrics** |  |  |  |
| The example datasets should be a subset of the ones already used for the EPT challenge. | 63% |  |  |
| One dataset simulated at 3 T (128 MHz) on a cylinder (with and without noise) should be available. | 68% |  |  |
| **One dataset simulated at** 3 T (**128 MHz) on a brain model + tumor inclusion (with and without noise) should be available.** | 79% | Yes |  |
| One dataset measured at 3 T (128 MHz) on a phantom should be available (reached consensus).  Statement: Such a phantom should be a sphere (diameter=12cm) and reference electrical properties measured using probes during phantom construction should be provided. | 58% |  |  |
| **Alongside the example datasets, ANALYSIS SCRIPTS to be run on the reconstructed EP maps should be provided.** | 79% | Yes |  |
| The analysis scripts can be written in multiple languages, but should include Matlab. | 68% |  |  |
| **The analysis scripts can be written in multiple languages, but should include Python.** | 79% | Yes |  |
| The analysis scripts should be supplied as precompiled executables for most major operating systems. The source code will also be provided. | 63% |  | removed |
| When possible, people should provide the results from the above scripts in the supplementary materials of their publications to allow direct comparisons between methodologies. | 63% |  |  |
| A standardized template should be provided to be used as supplementary materials in publications for such type of reporting, e.g., one image of the reconstructed EP maps, and a table with the quantitative results from the analysis scripts. | 63% |  |  |
| The standardized template and scripts should be provided as supplementary material of the standardization guideline. | 68% |  |  |
| **The following should be computed in these scripts for each tissue of interest: [Median]** | 79% | Yes |  |
| **The following should be computed in these scripts for each tissue of interest: [Interquartile range]** | 84% | Yes |  |
| **The following should be computed in these scripts for each tissue of interest: [Absolute error]** | 79% | Yes |  |
| **The following should be computed in these scripts for each tissue of interest: [Relative error]** | 89% | Yes |  |
| The following should be computed in these scripts for the whole volume (all tissues where electrical properties were reconstructed): [NRMSE] | 74% |  |  |
| The masks used for these analyses should not be eroded despite the risk that errors at tissue boundaries will skew the quantitative values. | 32% |  | removed |
| **The masks used for these analyses should contain different levels of erosion, and the results should be computed and reported for these different levels as in the EPT challenge (0-2-4 voxels erosion at boundaries).** | 84% | Yes |  |

**Supplementary table 4:** results Round 4.

| **Round 4 – 16 responders** | **Agreement** | **Consensus** | **Note** |
| --- | --- | --- | --- |
| **Color-maps** |  |  |  |
| Regions of negative electrical properties values should have the same color as the: [background] If one has a strong preference for one option instead of the other, please consider that in the assigned points to each option. | 69% |  | almost |
| Regions of negative electrical properties values should have the same color as the: [lower limit of the colorbar, this being >=0] If one has a strong preference for one option instead of the other, please consider that in the assigned points to each option. | 12% |  |  |
| In publications, the color-maps may have different colors than the recommended ones (authors' freedom); but maps using the recommended colors should also be displayed (e.g., in supporting materials of publications) to facilitate comparison between papers. | 56% |  |  |
| The color-map for conductivity should be: [exactly Lipari] | 44% |  |  |
| The color-map for conductivity should be: [warm-like Lipari] | 50% |  |  |
| The color-map for relative permittivity should be: [exactly Navia] | 37% |  |  |
| The color-map for relative permittivity should be: [cold-like Navia] | 50% |  |  |
| **Ranges** |  |  |  |
| **For scientific publications, the min-max range applied to a color-map should not be fixed to certain values, but should always be freely adaptable. Note that this means that two different papers showing conductivity maps, e.g. in the brain, may adopt two different max values, hence the color related to conductivity in the white matter may be different between the two papers. This may limit direct comparison between color-maps but can also help highlight details.** | 83% | Yes |  |
| For clinical publications, the min-max range applied to a color-map should not be fixed to certain values, but should always be freely adaptable. Note that this means that two different papers showing conductivity maps, e.g. in the brain, may adopt two different max values, hence the color related to conductivity in the white matter may be different between the two papers. This may limit direct comparison between color-maps but can also help highlight details. | 69% |  | almost |
| **In publications, the min-max ranges can be freely adaptable (authors' freedom); but maps using predefined min-max ranges should be displayed (e.g., in supporting materials of publications) to facilitate comparison between papers. These ranges will be therefore defined and will be different based on frequency and anatomical area.** | 75% | Yes |  |
| **The range for brain conductivity should be fixed: 0-2.5 S/m at** 3 T (**128 MHz) to facilitate comparison between publications.** | 75% | Yes |  |
| The range for brain relative permittivity should be fixed: 30-100 at 3 T (128 MHz) to facilitate comparison between publications. | 56% |  |  |
| **Data and Metrics** |  |  |  |
| The example datasets should be a subset of the ones already used for the EPT challenge. | 44% |  |  |
| One dataset simulated at 3 T (128 MHz) on a cylinder (with and without noise) should be available. | 63% |  |  |
| **One dataset measured at** 3 T (**128 MHz) on a phantom should be available (reached consensus).  Statement: Such phantoms should be: 1) a sphere (diameter=12cm) and 2) a cylinder (diameter= 12 cm length=12 cm); reference electrical properties values measured using probes should be provided.** | 75% | Yes |  |
| **Alongside the example datasets, analysis scripts to be run on the reconstructed ep maps should be provided (reached consensus). Statement: the analysis scripts can be written in multiple languages, but should include Matlab.** | 75% | Yes |  |
| When possible, people should provide the results from the above scripts in the supplementary materials of their publications to allow direct comparisons between methodologies. | 69% |  |  |
| A standardized template should be provided to be used as supplementary materials in publications for such type of reporting, e.g., one image of the reconstructed EP maps, and a table with the quantitative results from the analysis scripts. | 69% |  |  |
| The standardized template and scripts should be provided as supplementary material of the standardization guideline. | 63% |  |  |
| **The following should be computed in these scripts for the whole volume (all tissues where electrical properties were reconstructed):[NRMSE]** | 75% | Yes |  |

**References**

1. Fuderer M, Wichtmann B, Crameri F, et al. Color-map recommendation for MR relaxometry maps. Magn Reson Med. 2025;93:490-506. doi: 10.1002/mrm.30290.
2. Diamond IR, Grant RC, Feldman BM, et al. Defining consensus: a systematic review recommends methodologic criteria for reporting of Delphi studies. J Clin Epidemiol. 2014;67:401-409. doi:10.1016/j.jclinepi.2013.12.002
